# Rare case of gemination and fusion involving supernumerary teeth and second mandibular molar - Case report

**DOI:** 10.4317/jced.60865

**Published:** 2024-02-01

**Authors:** Vinícius-Canavarros Palma, João-Guilherme-Medeiros Leite, Letícia-Lopes Rocha, Mike-dos Reis Bueno, Talita-Nicaretta Canavarros, Luiz-Evaristo-Ricci Volpato

**Affiliations:** 1 School of Dentistry, University of Cuiabá, Cuiabá, MT, Brazil; 2 School of Dentistry, São Leopoldo Mandic, Campinas, SP, Brazil; 3 Department of Dentistry, Mato Grosso Cancer Hospital, Cuiabá, MT, Brazil

## Abstract

This article presents the rare case of a patient with gemination and fusion involving conical supernumerary teeth and the second mandibular molar, along with its treatment. The 13-year-old girl, without any associated syndromes and currently undergoing orthodontic treatment, was referred for evaluation due to the presence of a supernumerary tooth. Following a clinical examination and analysis of a panoramic radiograph, a cone beam computed tomography was requested for a more detailed assessment of the supernumerary tooth and its relationship with adjacent structures. The sagittal sections revealed the presence of two conical paramolar supernumerary teeth, which were geminated and fused to the mesial root of tooth 47. The suggested treatment was the surgical removal of the supernumerary teeth, which was successfully performed without complications and with good healing. After one-year post-procedure, the formation of periodontal ligament was verified at the site where the supernumerary teeth were extracted. The simultaneous occurrence of gemination of two supernumerary teeth and their fusion to a mandibular molar is extremely rare, and their surgical removal provided the patient with better plaque control in the region and contributed to the progress of orthodontic treatment.

** Key words:**Gemination and fusion of supernumerary teeth and second mandibular molar.

## Introduction

Abnormalities in the shape, position, structure, and number of teeth in an individual can occur because the process of dental germ development, both in primary and permanent dentition, is susceptible to genetic, systemic, and local factors interactions (1).

Supernumerary teeth are teeth formed in excess in addition to the normal series. They can have different sizes, shapes, and locations and can be found in any region of the dental arch (2). In primary dentition, they usually have a conical shape, while in permanent dentition, they can have various forms (3). Based on their location, they can be classified as mesiodens (located in the midline), paramolar (located buccally between the second and third molars), and distomolar (located distally to the third molar) (4).

In rare cases, supernumerary teeth can be fused with adjacent teeth. Fusion can involve enamel, dentin, cementum, pulp, or any of their canals. Fusion of supernumerary teeth involving molars is even rarer, usually occurring in the anterior region of the maxilla (5).

Gemination is a dental anomaly defined as the attempt of a single dental bud to divide, resulting in the formation of a large tooth with a bifid crown or two completely divided teeth with separate crowns and roots (6), sharing the same root canal (7). It is an uncommon condition in permanent dentition and extremely rare in the posterior region of the dental arch. Early diagnosis of this dental anomaly is important since it can affect occlusal harmony (8), leading to issues in orthodontic planning and treatment (9).

Treatment depends on the type, position, and potential complications that can be clinically identified and through imaging examinations, although surgical removal is the most common treatment option (10).

This study presents a rare case of a patient with gemination and fusion involving conoid supernumerary teeth and the second mandibular molar, along with its treatment.

## Case Report

A 13-year-old Caucasian female patient, with normal facial characteristics and no associated syndromes, undergoing orthodontic treatment and without any complaints of pain, was referred by the orthodontist for evaluation due to the presence of a supernumerary tooth.

**Figure 1:**
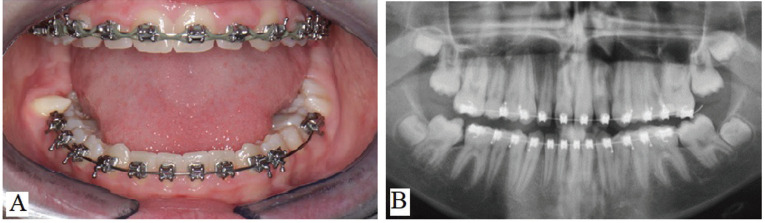
A) Initial clinical aspect of the patient, showing permanent dentition under orthodontic treatment and the presence of a conoid supernumerary tooth located buccally to tooth 47. B) Panoramic radiograph of the patient demonstrating the presence of all erupted permanent teeth, teeth in the process of eruption, or intraosseous formation. The presence of the supernumerary tooth can be observed overlapping the image of tooth 47.

During the clinical examination and analysis of the panoramic radiograph previously taken by the patient, the presence of a conoid supernumerary paramolar tooth was observed, located near the mesio-buccal cusp of the right second mandibular molar (Fig. 1).

The patient was requested to undergo cone beam computed tomography (CBCT) in order to evaluate the three-dimensional location of the supernumerary tooth and its relationship with nearby structures such as the second molar and the inferior alveolar nerve.

**Figure 2:**
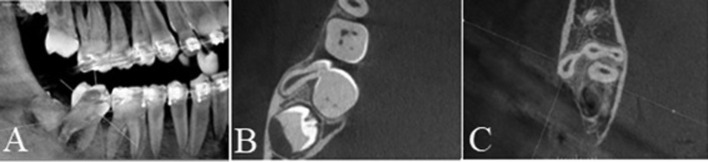
A) Sagittal section of the CBCT showing the presence of two conoid supernumerary teeth near tooth 47, with the crown of the first tooth facing occlusally and the second tooth impacted, tilting mesially toward the cervical line of tooth 47. The impaction of the germ of tooth 48 can also be visualized. B) Axial section highlighting the gemination between the two supernumerary teeth with a single root canal and fusion with tooth 47. C) Axial section showing that the fusion also involved the distal root of tooth 47.

On the sagittal sections, the presence of two conoid paramolar supernumerary teeth was observed, both located buccally to tooth 47. One tooth was in a vertical position and partially erupted, while the second tooth was impacted, tilted mesially, with the crown close to the cervical line of tooth 47. Analysis of the axial sections revealed that the supernumerary teeth were geminated, and the root portion of the tilted supernumerary tooth was fused to the mesial root of tooth 47 (Fig. 2).

After analysing the CBCT, the proposed treatment suggestion was the surgical removal of the supernumerary teeth. Due to the diagnosis of fusion between the supernumerary teeth and tooth 47, the plan was to section the teeth and remove the unfused parts first, followed by the removal of the fused portion.

The extractions began with the blockage of the inferior alveolar nerve, lingual nerve, and buccal nerve, following the necessary aseptic conditions. After the anesthetic blockage, the extractions were performed in stages. Odontosection and removal of the coronal portion of the vertically positioned supernumerary tooth were carried out. After removal, an area of demineralized white spots was visible on the buccal surface of tooth 47, likely related to difficulty maintaining hygiene in that region. Subsequently, the other supernumerary tooth was sectioned to release the entire structure of the two teeth that were not fused to tooth 47. The fused portion of the supernumerary tooth was then removed using a high-speed diamond bur. The germ of tooth 48 was also removed, and suturing was performed.

For the postoperative period, the patient was recommended to use 0.12% chlorhexidine mouthwash, anti-inflammatory drugs, and analgesics. Follow-up appointments at seven, 12, and 100 days showed good healing without postoperative complications.

**Figure 3:**
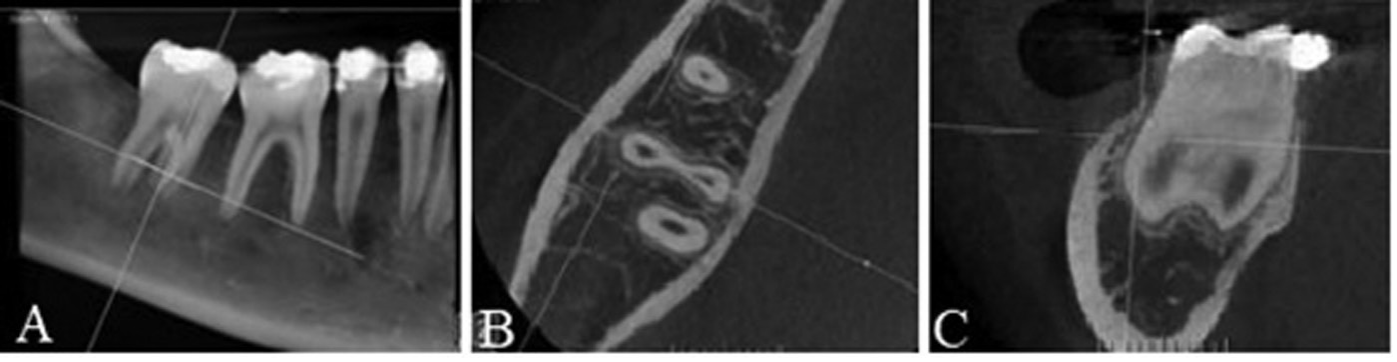
Follow-up CBCT of the case after one year, where it can be seen in A) Sagittal section, good bone healing in the extraction sites; B) Axial section, visualization of the periodontal space in the buccal portion of the mesial root of tooth 47, where the fusion of the supernumerary teeth was present; C) Confirmed in the coronal section.

The CBCT performed one year after the surgical procedure demonstrated good bone healing in the region where the supernumerary teeth were removed, as well as the formation of periodontal ligament in the buccal portion of the mesial root of tooth 47, where the fusion of the supernumerary teeth was present (Fig. 3).

## Discussion

The present report describes the rare case of a patient with two supernumerary dysmorphic teeth that were geminated and fused to tooth 47. These teeth were extracted due to their anomalous condition and position in the dental arch, and the patient’s follow-up after one year.

The development of teeth beyond the normal series, known as supernumerary teeth, occurs more frequently in male patients (4) and is often associated with syndromes (1). However, in this reported case, it involved a non-syndromic female patient.

In non-syndromic patients, the occurrence of more than one supernumerary tooth is uncommon (10). These teeth are usually located in the maxilla, especially in the midline (2). This case stands out as it presents two supernumerary teeth located in the posterior region of the mandible.

Supernumerary teeth can either erupt in the oral cavity or remain impacted (11). In this case, both teeth were dysmorphic, which is a common presentation of supernumerary teeth (12). One of the supernumerary teeth that was partially erupted has a conoid shape, while the other, impacted tooth has a rudimentary shape.

The patient was requested to undergo a CBCT scan for a better evaluation of the location of the supernumerary tooth and adjacent structures. CBCT is an imaging examination that allows three-dimensional visualization of anatomical structures in various planes and depths (13). Through this examination, it was possible to observe that there were two geminated and fused supernumerary teeth with tooth 47, which is a rare situation. The fusion of supernumerary teeth with normal teeth is uncommon, and when it occurs, it is more frequent in the anterior region of the maxilla. Reports involving the fusion of supernumerary teeth with lower molars are even rare (5).

The prevalence of geminated supernumerary teeth is 0.1% in permanent dentition (14). The occurrence of supernumerary teeth with altered shapes is an even rarer phenomenon (5), especially in the posterior region of the mandible (6).

The suggested treatment was the surgical removal of the supernumerary teeth because their location and positioning made hygiene and proper occlusion difficult for the patient. The presence of supernumerary teeth was possibly causing increased biofilm retention, leading to gingival inflammation and enamel demineralization, clinically observed as an active white spot on the buccal surface of tooth 47.

To prevent post-operative complications in cases of impacted supernumerary tooth extraction, periodic follow-up evaluations are essential (5).

The post-surgical follow-up of this case showed good clinical progress, which was further confirmed by the CBCT scan at the one-year control, where the periodontal space in the fusion region of the supernumerary tooth was visualized.

## Conclusions

The presence of two supernumerary dysmorphic teeth in the region of the permanent lower second molar in a non-syndromic patient is rare, as is their gemination and fusion with the normal series tooth. This diagnosis, as well as the determination of the appropriate treatment plan, were only possible with the use of CBCT. The surgical removal of the supernumerary teeth allowed the patient to achieve better biofilm control in the area and facilitated the proper management of orthodontic treatment.

## References

[1] Suljkanovic N, Balic D, Begic N (2021). Supernumerary and Supplementary Teeth in a Non-syndromic Patients. Med Arch.

[2] Salcido-García JF, Ledesma-Montes C, Hernández-Flores F, Pérez D, Garcés-Ortíz M (2004). Frequency of supernumerary teeth in Mexican population. Med Oral Patol Oral Cir Bucal.

[3] Wang XP, Fan J (2011). Molecular genetics of supernumerary tooth formation. Genesis.

[4] Ata-Ali F, Ata-Ali J, Peñarrocha-Oltra D, Peñarrocha-Diago M (2014). Prevalence, etiology, diagnosis, treatment and complications of supernumerary teeth. J Clin Exp Dent.

[5] Zhu M, Liu C, Ren S, Lin Z, Miao L, Sun W (2015). Fusion of a supernumerary tooth to right mandibular second molar: a case report and literature review. Int J Clin Exp Med.

[6] Soin A, Sharma G, Soin G, Raina A, Mutneja P, Nagpal A (2015). Multiple Geminated Supernumerary Premolars: A Rare Case Report. Case Rep Dent.

[7] Mahendra L, Govindarajan S, Jayanandan M, Shamsudeen SM, Kumar N, Madasamy R (2014). Complete bilateral gemination of maxillary incisors with separate root canals. Case Rep Dent.

[8] Sandeep S, Kumar GJ, Potdar S, Bhanot R, Vathare AS, Tiwari RVC (2020). Rare case of gemination of mandibular third molar-A case report. J Family Med Prim Care.

[9] Altug-Atac AT, Erdem D (2007). Prevalence and distribution of dental anomalies in orthodontic patients. Am J Orthod Dentofacial Orthop.

[10] Borges AH, Siqueira CR, Pedro FL, Palma VC, Sakai VT, Volpato LE (2013). Growth hormone insensitivity syndrome: unusual oral manifestations. J Dent Child (Chic).

[11] Parolia A, Kundabala M, Dahal M, Mohan M, Thomas MS (2011). Management of supernumerary teeth. J Conserv Dent.

[12] Kumar A, Namdev R, Bakshi L, Dutta S (2012). Supernumerary teeth: Report of four unusual cases. Contemp Clin Dent.

[13] Bueno MR, Estrela C, Granjeiro JM, Estrela MRA, Azevedo BC, Diogenes A (2021). Cone-beam computed tomography cinematic rendering: clinical, teaching and research applications. Braz Oral Res.

[14] Sandeep S, Kumar GJ, Potdar S, Bhanot R, Vathare AS, Tiwari RVC (2020). Rare case of gemination of mandibular third molar-A case report. J Family Med Prim Care.

